# Effects of damping-off caused by *Rhizoctonia solani* anastomosis group 2-1 on roots of wheat and oil seed rape quantified using X-ray Computed Tomography and real-time PCR

**DOI:** 10.3389/fpls.2015.00461

**Published:** 2015-06-24

**Authors:** Craig J. Sturrock, James Woodhall, Matthew Brown, Catherine Walker, Sacha J. Mooney, Rumiana V. Ray

**Affiliations:** ^1^School of Biosciences, University of Nottingham, Sutton Bonington Campus, LoughboroughUK; ^2^The Food and Environment Research Agency, Sand HuttonUK

**Keywords:** *Rhizoctonia solani*, X-ray Computed Tomography, qPCR, wheat, oil seed rape, fungi, soil

## Abstract

*Rhizoctonia solani* is a plant pathogenic fungus that causes significant establishment and yield losses to several important food crops globally. This is the first application of high resolution X-ray micro Computed Tomography (X-ray μCT) and real-time PCR to study host–pathogen interactions *in situ* and elucidate the mechanism of *Rhizoctonia* damping-off disease over a 6-day period caused by *R. solani*, anastomosis group (AG) 2-1 in wheat (*Triticum aestivum* cv. Gallant) and oil seed rape (OSR, *Brassica napus* cv. Marinka). Temporal, non-destructive analysis of root system architectures was performed using RooTrak and validated by the destructive method of root washing. Disease was assessed visually and related to pathogen DNA quantification in soil using real-time PCR. *R. solani* AG2-1 at similar initial DNA concentrations in soil was capable of causing significant damage to the developing root systems of both wheat and OSR. Disease caused reductions in primary root number, root volume, root surface area, and convex hull which were affected less in the monocotyledonous host. Wheat was more tolerant to the pathogen, exhibited fewer symptoms and developed more complex root systems. In contrast, *R. solani* caused earlier damage and maceration of the taproot of the dicot, OSR. Disease severity was related to pathogen DNA accumulation in soil only for OSR, however, reductions in root traits were significantly associated with both disease and pathogen DNA. The method offers the first steps in advancing current understanding of soil-borne pathogen behavior *in situ* at the pore scale, which may lead to the development of mitigation measures to combat disease influence in the field.

## Introduction

*Rhizoctonia solani* Kühn (teleomorph = *Thanatephorus cucumeris* Donk) is a ubiquitous soil-borne plant pathogenic fungus which causes significant yield losses in many agriculturally important crops (Verma, 1996; Paulitz et al., 2006). Individual isolates of *R. solani* are classified into anastomosis groups (AGs) based on their hyphal incompatibility and their host specificity (Anderson, 1982). For example, AG2-1 and AG4 are associated with stem and root rot diseases in dicotyledonous crop species belonging to *Brassicaceae* (Gugel et al., 1987; Sneh et al., 1991; Tewoldemedhin et al., 2006) whilst isolates of AG8 cause ‘bare patch’ or root rot on monocotyledonous crops from *Poaceae* (Paulitz et al., 2002).

The predominant population of *R. solani* causing severe seedling diseases associated with establishment losses of up to 80–100% and final yield loss of up to 30% of oil seed rape (OSR, *Brassica napus*) worldwide belongs to AG2-1 (Tahvonen et al., 1984; Kataria and Verma, 1992; Khangura et al., 1999). Highly virulent isolates of AG2-1 cause pre- and post-emergence damping-off, stem, and root rot with characteristic water soaked lesions on the root and hypocotyl, stunting of plant growth, root necrosis and cortex tissue maceration, and subsequent death in OSR (Yang et al., 1992). Recent soil surveys, carried out in USA (Schroeder et al., 2011) and UK (Brown et al., 2014) on fields growing winter wheat (*Triticum aestivum*) have revealed the most common pathogen present in soils of increased rotational frequency with OSR is *R. solani* AG2-1, shown in >69% of fields (*n* = 90) in England.

Whilst the pathogenicity and aggressiveness of AG2-1 to OSR have been previously studied (Yitbarek et al., 1987; Kranz, 1988), less is known of the impact of this group of pathogens on wheat roots. AG2-1 isolates have been shown to be pathogenic to cereals to varying degrees. Tewoldemedhin et al., (2006) reported AG2-1 isolates were weakly pathogenic to barley and wheat roots. In contrast, Roberts and Sivasithamparam (1986) reported AG2-1 isolates from wheat roots in ‘bare patch’ in Western Australia were highly pathogenic to wheat causing an 80% disease index which was similar to disease caused by AG8 isolates. Thus, at present, the ability of AG2-1 to cause significant damage to the root system of seedlings of monocotyledonous crops such as wheat remains unclear.

The etiology of soil-borne diseases caused by pathogens such as *R. solani* on plant seeds and roots below ground has until recently been difficult to study. Traditionally, assessment of disease incidence and severity has involved the use of visual observations of symptoms of infection on affected plant organs following the physical extraction of plants from the ground (Kranz, 1988). However, the inherently destructive nature of visual disease inspection means that it is not possible to monitor temporal disease development and effects on root traits and system architecture. Furthermore, destructive sampling in the field often results in an incomplete root system extraction and loss of the most severely infected or severed primary/secondary roots.

Non-destructive methods for imaging plant roots *in situ* in soil, such as X-ray micro Computed Tomography (X-ray μCT), have become an important tool for quantifying plant root system architecture development in three dimensions (see review by Mooney et al., 2012). However, to date the application of X-ray μCT to investigate the impact of root rot pathogens has been relatively limited to Han et al. (2008) who studied the effects of common potato scab caused by *Streptomyces scabies* on tubers in soil. This was the first use of medical X-ray CT in a phytopathological study to successfully segment root structures from CT images and demonstrated diseased plants had significantly less complex root systems, in addition to delayed root growth and branching. A subsequent study by the same researchers using CT showed the effects of common potato scab on the density of seed and peripheral organs of potato plants in soil over a 10 week period (Han et al., 2009). Interestingly, an early application of a medical CT system to soil science by Grose et al. (1996) measured moisture content in bulk soil and in the soil around roots to predict suitable growth conditions for both *R. solani* and *Gaeumannomyces graminis*. Although at relatively coarse resolutions (200 μm) compared to the resolution achievable on modern systems for similar sized pots (6 cm diameter), the study successfully quantified heterogeneous moisture gradient in the vicinity of the plant roots and demonstrated the potential of the technique for investigation of environmental factors on the soil–plant–microbe system. Recent advances in the sensitivity of X-ray detectors within industrial μCT systems have facilitated much faster acquisition times (minutes rather than hours) facilitating easier repeated scanning of the same sample to visualize the temporal dynamics of plant root systems in undisturbed soil a (Tracy et al., 2012; Zappala et al., 2013b)

Microbiological methods for detection and quantification of target AGs of *R. solani* in soil are highly labor intensive and time consuming, involving the use of soil baiting methods that are often inefficient in detecting and isolating *R. solani*, and microscopy (Sneh et al., 1991). Furthermore, low population densities of *R. solani* in the soil and the lack of selective isolation media for the species make quantification difficult and unreliable. In the last decade, several conventional or real-time quantitative polymerase chain reaction (qPCR) assays have become an established tool for rapidly quantifying fungal pathogens including targeted AGs of *R. solani* at low detection limits in both soil and infected plant tissues (Filion et al., 2003a,b; Sayler and Yang, 2007; Okubara et al., 2008; Budge et al., 2009; Woodhall et al., 2013). We propose that the combination of these two powerful techniques, qPCR and X ray μCT, can allow improved new insight into the temporal host–pathogen interactions and provide quantitative data on the impact of soil-borne pathogens on root architectural systems of crop plants grown in soil. The main aim of this study was to elucidate the mechanism of disease caused by AG2-1 of *R. solani* on root traits and system architecture of two different crops, the monocot, wheat, and the dicot, OSR.

## Materials and Methods

### Soil, Plant, and Inoculum Preparation

The experiment was designed as a factorial block with two main factors, host and inoculation with two levels. The host crops were wheat, (*Triticum aestivum* cv. Gallant) or OSR (*Brassica napus* cv. Marinka) which were either non-inoculated or inoculated with *R. solani* AG2-1 (Isolate 159/8, Goll et al., 2014). The isolate was previously determined to be weakly pathogenic to wheat and pathogenic to OSR. There were nine replicates of the treatment combinations resulting in a total of 36 columns.

Soil columns (30 mm diameter × 70 mm length) were uniformly packed to a bulk density of 1.1 Mg m^-3^ with a Newport series loamy sand soil (sand 72.6%, silt 13.2%, and clay 14.2%; pH 6.35; organic matter 2.93%) collected from the University of Nottingham farm at Bunny, Nottinghamshire, UK (52.52°N, 1.07°W). Prior to packing, the soil was air-dried, sieved to <2 mm and sterilized by γ-irradiation at 27 kGy (Isotron, Daventry, UK). The pathogen treated soils were inoculated with five, 5-mm diameter plugs of actively growing *R. solani* mycelium equally distributed in the vertical direction of the soil during packing of the columns. Seeds of cv. Gallant and cv. Marinka were pre-germinated for 48 h on moist filter paper in petri dishes before being planted at 10 and 5 mm below the soil surface, respectively. The columns were then saturated, drained for 2 days (to a notional field capacity which represents the moisture content of the soil after free drainage had ceased) and placed in a growth room under conditions of 14°C day/night with an 8 h photoperiod and a photosynthetic photon flux density (PPFD) at plant level of 1000 μmol m^-2^ s^-1^. A transparent plastic unheated seed propagator was used to maintain high relative humidity levels and avoid surface drying of the soil during seedling establishment in the growth room. Three replicates for each treatment combination were randomly selected and destructively harvested via root washing and scored for disease 2, 4, and 6 days following inoculation (dfi). Root disease severity was assessed at each destructive sampling point on soil-free plants on scales from 0 to 5; 0 = no lesions, clean roots; 1 = small lesion on tap root; 2 = necrosis of upto 30%; 3 = necrosis covering 31–60% of the tap root; 4 = necrosis covering 61–99% of the tap root; 5 = completely severed tap root (Khangura et al., 1999). In addition, the three replicates selected for harvest at 6 dfi were also scanned using X-ray μCT at 2, 4, and 6 dfi to permit non-destructive quantification of root system development. Root architecture of the washed roots was assessed using WinRHIZO^®^ 2002c scanning equipment and software on each harvest day. The images collected were used to compare with the X-ray μCT images. Soil from the columns was further used for DNA extraction and pathogen quantification.

### X-Ray Micro Computed Tomography (μCT)

The replicate subset allocated for destructive sampling at 6 dfi (12 columns), were scanned at 2, 4, and 6 days using a Phoenix Nanotom^®^ (GE Measurement & Control Solutions, Wunstorf, Germany) X-ray μCT scanner. The scanner consists of a 180 kV nanofocus X-ray tube fitted with a tungsten transmission target and a 5-megapixel (2304 × 2304 pixels, 50 × 50 μm pixel size) flat panel detector (Hamamatsu Photonics KK, Shizuoka, Japan). A maximum X-ray energy of 110 kV, 140 μA current and a 0.15 mm thick copper filter was used to scan each sample which consisted of 1300 projection images acquired over a 360°rotation. Each projection image was the average of three images acquired with a detector exposure time of 500 ms in ‘Fast CT mode.’ The resulting isotropic voxel edge length was 19 μm (i.e., spatial resolution) and total scan time was 35 min. The total X-ray dose for each sample was calculated as 25.2 Gy over the three scans, which is below the 33 Gy threshold reported by Johnson (1936) which no detrimental effects of post-germination plant growth following exposure to X-ray radiation were observed (Zappala et al., 2013a). Reconstruction of the projection images was performed using the software datos| rec (GE Measurement & Control Solutions, Wunstorf, Germany) to produce 3-D volumetric data sets with dimension 30 × 30 mm (diameter × depth).

### Image Processing and Analysis

Plant root systems were non-destructively segmented using the *Region Growing* selection tool in VG StudioMAX^®^ 2.2 software as described by Tracy et al. (2012). To summarize, the *Region Growing* tool, allows the user to select connected structures within the data that have the same distribution of X-ray attenuation based on their gray values. The user assigns all root material to a region of interest which is then extracted as a separate binary image stack for measurement of root system architecture in RooTrak software. RooTrak software (Mairhofer et al., 2012) permits quantification of descriptive traits on root system architecture, such as total volume, surface area, maximum length and width, convex hull (relates to the space filling in 3D of an object), and centroid Z (relates to the center of mass of a 3D object). Due to small scales differences in seed depth in the reconstructed volumetric data, the measurement field of view was standardized to 30 mm × 25.80 mm (diameter × depth). Therefore, the maximum possible value for root length measurements is limited to 25.80 mm.

Soil porosity (total and incremental with depth) was quantified in FIJI image analysis software (Schindelin et al., 2012) using a modified method of Tracy et al. (2012). To summarize, a resized 16 bit image stack of dimensions 17.1 mm × 17.1 mm × 19 mm (900 × 900 pixels × 1000 images) was first prepared to exclude the area outside of the soil column (i.e., the container and the surrounding air space). Images were binarised to define the air filled pore space with a value of 0 and the ‘solid’ soil with a value of 1 using the isodata threshold algorithm which performed the best in an evaluation study. Soil porosity for each slice image was calculated based on the percentage of air to the total volume of the resized stack.

### Real Time Quantitative PCR for AG2-1 of *R. solani*

DNA was extracted from soil as described in Woodhall et al. (2012), except sample size was reduced to 45 g and then added to a 250 ml Nalgene bottle with 3 ml antifoam B with six 25.4 mm stainless steel ball bearings and 90 ml grinding buffer (120 mM sodium phosphate buffer pH 8, 2% cetrimonium bromide, 1.5 M sodium chloride). Real-time PCR was undertaken using a 7500 real-time PCR system. Environmental Master Mix 2.0 (Life Technologies, USA) was used for all real-time PCR and consisted of half the total reaction volume of 25 μl, whilst 5 μl consisted of the DNA sample. Primers (MWG Biotech, Germany) and hydrolysis probe specific for AG2-1 (Budge et al., 2009) were used and added to a final concentration in the reaction of 300 and 100 nM, respectively, with the remaining volume made up with molecular grade water. Cycling conditions consisted of 50°C for 2 min, 95°C for 10 min, and 40 cycles of 95°C for 15 s, and 60°C for 1 min. Each sample was tested in duplicate and an average Ct-value was determined. Target DNA in soil samples was quantified by including six DNA standards on each PCR run. The standards consisted of a DNA sample of known concentration taken from culture of AG2-1 (Isolate 2023, Food and Environment Research Agency, UK) which was used to produce a dilution series of five 10-fold dilutions. The amount of DNA was then determined by linear regression.

### Statistical Analysis

Root growth and architecture traits were analyzed using analysis of variance (ANOVA) for repeated measures and corrected for degrees of freedom for all time related effects with Greenhouse–Geisser Epsilon factor. Architecture traits were root volume, surface area, convex hull volume, maximum width, and length. Pathogen DNA data were analyzed by ANOVA containing sampling time, crop, and inoculation as interacting factors in the treatment structure. Regression analysis was used to investigate the relationships between root traits, disease score, and pathogen DNA, using a simple linear model for each crop separately. All analyses were performed in Genstat 15, version 15.1.0.8035.

## Results

### Disease Development and Pathogen DNA Accumulation in Soil

**FIGURE 1:**
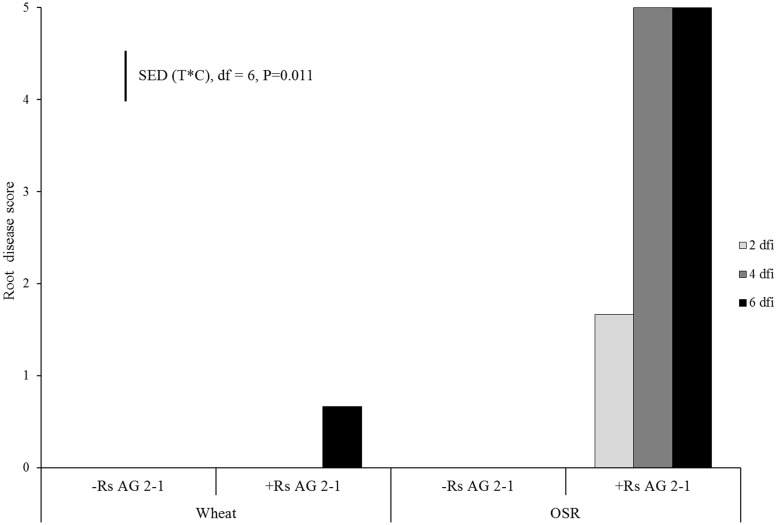
**Disease severity (0 = no lesions, clean roots; 1 = small lesion on tap root; 2 = necrosis of up to 30%; 3 = necrosis covering 30–60% of the tap root; 4 = necrosis covering 61–99% of the tap root; 5 = completely severed tap root) assessed 2 days following inoculation (dfi), 4 and 6 dfi on wheat and oil seed rape (OSR) plants inoculated with *Rhizoctonia solani* AG2-1 (Rs AG2-1)**. disease symptoms were shown in the control treatment for both crops. Bar shows standard error of difference (SED) for the interaction between sample time (T) at 2, 4, or 6 dfi and crop (C) species (wheat or OSR).

No symptoms of root disease were observed in the non-inoculated treatments (control) for either crop species (**Figure 1**). OSR plants developed visible lesions on roots as soon as 2 dfi. The symptoms rapidly progressed from moderate (necrosis covering 31–60% of the root, disease score 3) to severe (completely severed taproot, disease score 5) by 4 dfi resulting in complete maceration of root tissue by day 6 (**Figure 1**). Wheat plants exhibited significantly lower disease severity compared to OSR plants (*P* = 0.011), with symptoms classified as slight (small lesions on the primary roots, disease score 1) which were first detected at 6 dfi (**Figure 1**).

**FIGURE 2:**
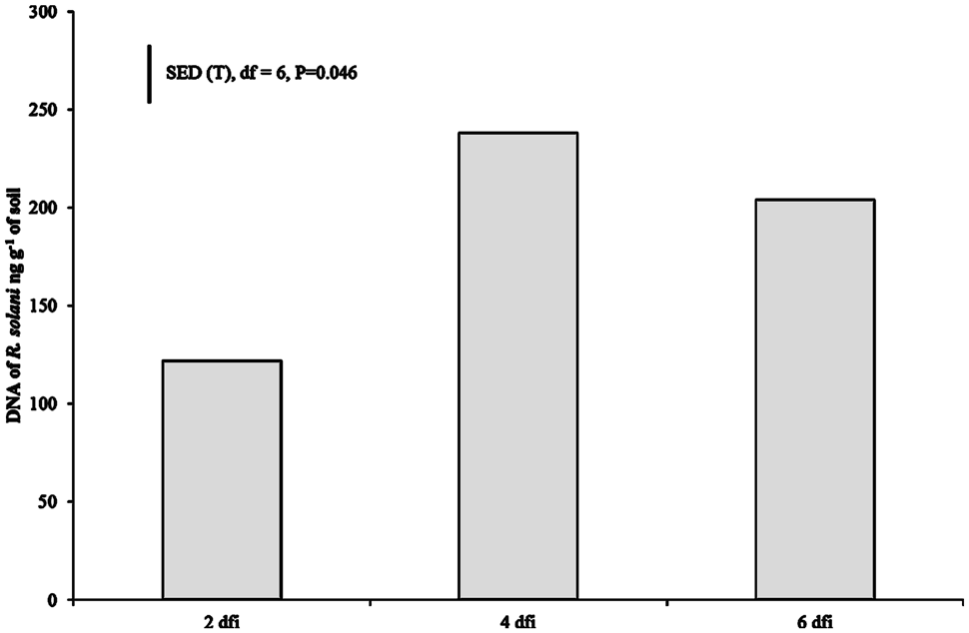
**Pathogen DNA quantified using real-time PCR at 2, 4, and 6 dfi from soil inoculated with *R. solani* AG2-1 (Rs AG2-1).** Bar shows SED for sample time (T) for both crop species.

DNA of *R. solani* was not detected in the soil of non-inoculated plants at 2 dfi, but was quantifiable at 4 and 6 dfi at low concentrations (0.008 and 0.019 ng g^-1^) in two soil columns. In contrast, DNA in inoculated soils of both crops at 2 dfi was above 100 ng g^-1^ (**Figure 2**). The trend of DNA accumulation over the duration of the sampling period was similar for the two crops showing an increase in pathogen DNA by day 4 followed by a plateau by 6 dfi (**Figure 2**). The mean pathogen DNA in the OSR treatment at 4 dfi was approximately 45% higher than in the wheat treatment (*P* = 0.063) although no differences were observed between crops for 2 or 6 dfi.

### Impact of *R. solani* AG2-1 on Root System Architecture of Wheat and OSR

**FIGURE 3:**
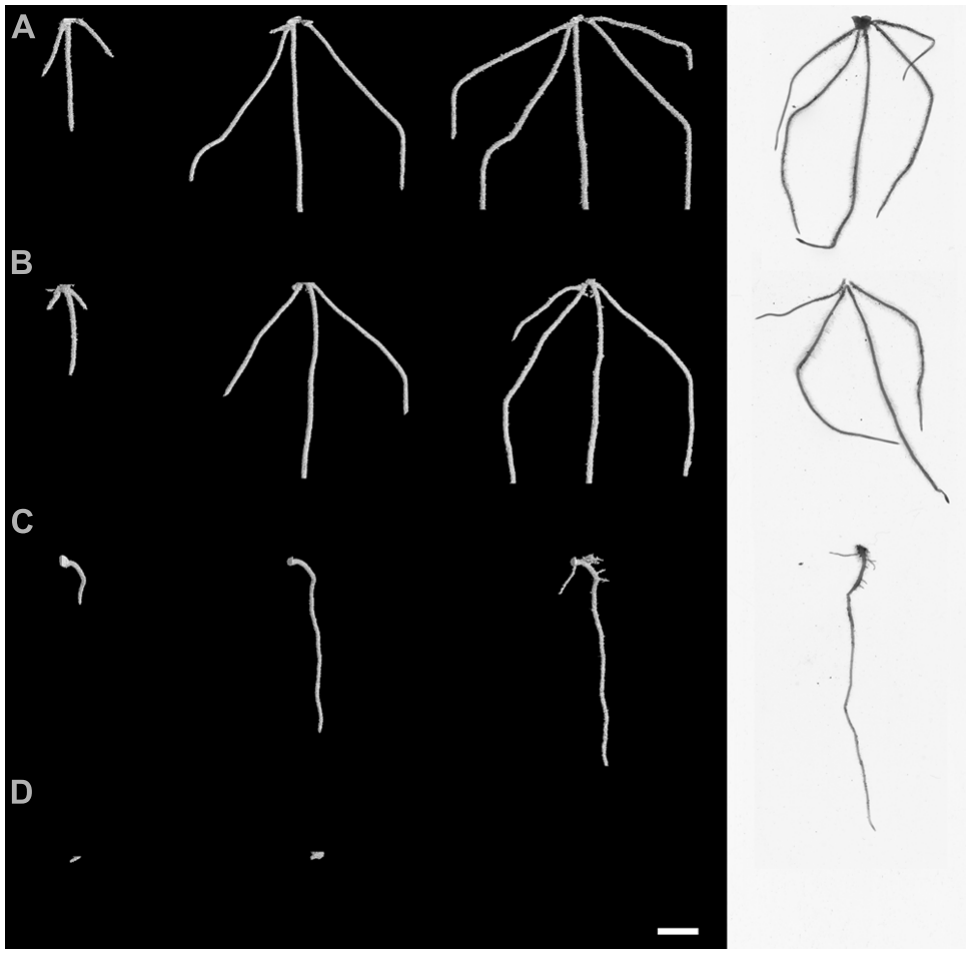
**Root system architecture at 2, 4, and 6 dfi visualized non-destructively by X-ray micro Computed Tomography (X-ray CT) and at 6 dfi by destructive WinRHIZO^®^ (white background) for control and *R. solani* AG2-1 treated wheat **(A,B)** and OSR plants **(C,D)**.** Scale bar = 5 mm.

Visual assessment of X-ray μCT 3D images and WinRHIZO^®^ images suggested major differences in root system architecture under the experimental factors, inoculation and crop (**Figure 3**; Supplementary Videos 1 and 2). Control OSR plants had a characteristic single tap root that developed lateral roots by 6 dfi. Typically, wheat plants developed between 3 and 5 primary roots with no lateral roots by the end of the experiment. Initial root growth of OSR plants was inhibited in soils inoculated with AG2-1 of *R. solani* and resulted in complete maceration of root tissue by 6 dfi. Disease effects were less obvious on wheat roots from inoculated soils with *R. solani* (**Figure 3**).

**FIGURE 4:**
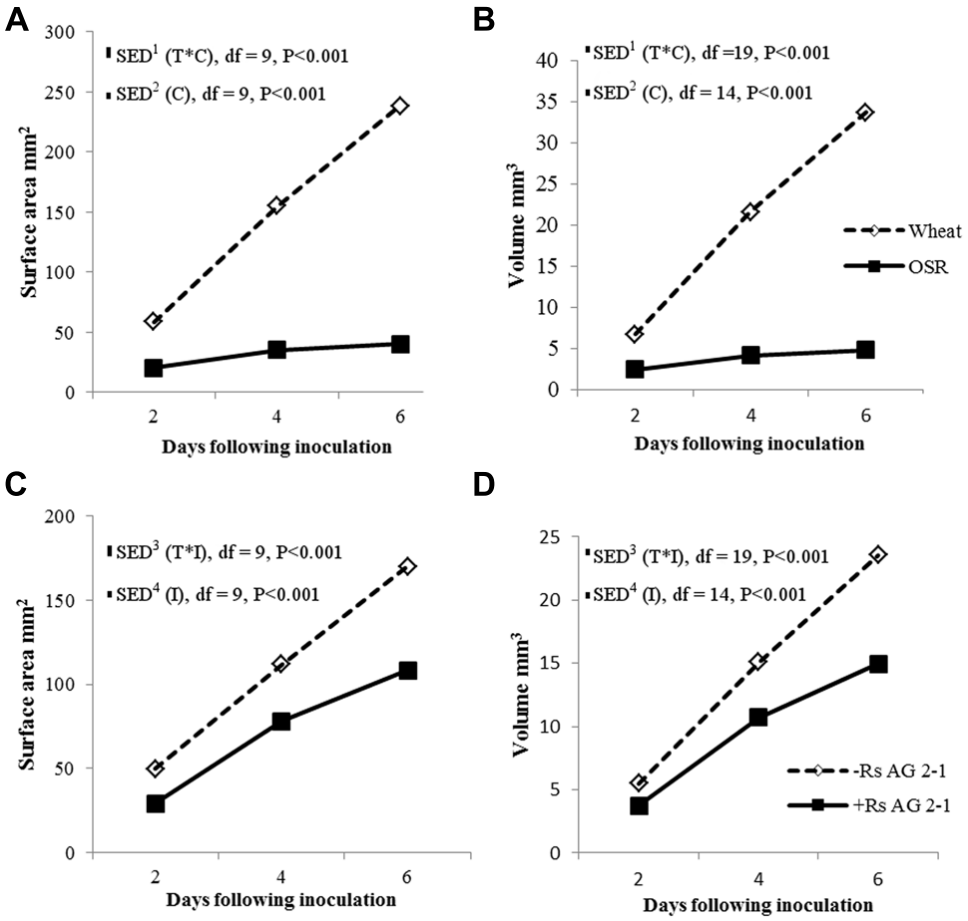
**Root system volume and surface area over time (T) for crop (C), **(A,B)** and inoculation (I) with *R. solani* AG2-1 **(C,D)**.** Interactions for surface area and volume were detected using repeated measures ANOVA with degrees of freedom (df) corrected by Greenhouse–Geisser epsilon factor. Bars show SED for (1) comparing means for treatment combinations; (2) comparing means with the same level of C; (3) for comparing means for the same level of I and species (wheat or OSR); (4) comparing means with the same level of I.

There were significant temporal differences for root volume and surface area measured using X-ray μCT between crops (**Figures 4A,B**; *P* < 0.001) and between inoculated and non-inoculated plants (**Figures 4C,D**; *P* < 0.001). The absence of interactions between crop and inoculation suggested root volume and surface area were affected mainly by intrinsic differences in root system characteristics of individual crop species and the presence of the pathogen in the soils. Inoculation significantly reduced root volume and surface area in both crops, however, the effects were greater in OSR, where these traits were affected immediately following inoculation and there were relatively small changes over time in trait parameters (**Figure 4**).

Root system traits for which significant temporal interactions between crop and inoculation were detected are shown in **Table 1**. The root system of wheat increased in length and width in time, despite inoculation, to a maximum of 25.8 and 29.3 mm, respectively (**Table 1**). A similar trend was observed for the control OSR plants with the root system length and width reaching 25.8 and 13.5 mm, respectively, by the end of the experiment. However, for the OSR plants inoculated with *R. solani*, root growth was inhibited from day 2, slight increases in length and width were observed by day 4 but ultimately at 6 dfi roots of inoculated plants were 96% shorter and 78% thinner than the controls (0.97 and 2.90 mm, respectively).

**Table 1:** Means for root system traits measured using X-ray micro Computed Tomography (X-ray CT) for incubation time, crop, and inoculation.

	Root max length (mm)				Root max width (mm)				Centroid Z (mm)				Convex hull (mm^3^)			
	Wheat		Oil seed rape (OSR)		Wheat		OSR		Wheat		OSR		Wheat		OSR	
Time (dfi)	Control	AG2-1	Control	AG2-1	Control	AG2-1	Control	AG2-1	Control	AG2-1	Control	AG2-1	Control	AG2-1	Control	AG2-1
2	9.88	11.13	7.97	1.20	13.87	5.61	10.42	1.85	6.44	8.01	5.46	1.07	128	49	30	2
4	25.80	25.80	23.00	1.27	27.63	25.43	13.18	3.77	16.04	16.47	15.65	1.03	1815	729	156	7
6	25.80	25.80	25.80	0.97	28.66	29.39	13.50	2.90	14.86	14.50	18.52	0.69	4123	2038	416	7
	SED	df			SED	df			SED	df			SED	df		
A	1.64	(16)			2.73	(10)			1.49	(16)			243	(12)		
B	1.53	(10)			1.76	(10)			1.45	(16)			244	(7)		
Effects	P				P				P				P			
T	0.001				0.001				0.001				0.001			
T × C	0.002				0.001				0.066				0.001			
T × I	0.001				0.176				0.002				0.009			
T × C × I	0.001				0.041				0.010				0.048			
GGE	0.6906				0.6699				0.6952				0.5052			

*dfi, days following inoculation; T, time; C, crop; I, inoculation; GGE, Greenhouse–Geisser epsilon; SED, standard error of difference; df, number of degrees of freedom.*

*A: SED for comparing means for with different levels of inoculation and crop, B: SED for comparing means with the same level of inoculation and crop (C × I) for different times (dfi) of measurement.*

Both inoculation treatments in wheat displayed a significant increase in centroid Z (an indication of root structure with depth) after 4 days incubation with a mean value of 16.04 and 16.47 mm for the control and inoculated plants, which then reduced to 14.86 and 14.5 mm, respectively, after 6 dfi. Control OSR plants displayed a sustained increase in centroid Z from 1.07 mm at 2 dfi to 18.52 mm at 6 dfi. Centroid Z remained consistently low throughout the experiment for the *R. solani* treated OSR plants (1 mm). (**Table 1**; time × crop × inoculation; *P* = 0.010.)

Convex hull (an indication of the volume of soil explored) increased in all treatments except in OSR inoculated plants, where it remained the same after 4 dfi and for wheat was significantly higher compared to OSR (*P* = 0.001). Inoculation with *R. solani* resulted in smaller rates of increase in convex hull in both plants (**Table 1**). The control wheat treatment showed a significantly higher convex hull which was almost twice the volume compared to the *R. solani* inoculated treatment with values of 4123 and 2038 mm^3^, respectively, after 6 dfi. The control OSR had a lower convex hull compared to wheat with a mean of 413 mm^3^. *R. solani* treated OSR exhibited the lowest convex hull with a mean of 7 mm^3^ remaining the same at 4 and 6 dfi (**Table 1**; time × crop × inoculation; *P* = 0.048).

**FIGURE 5:**
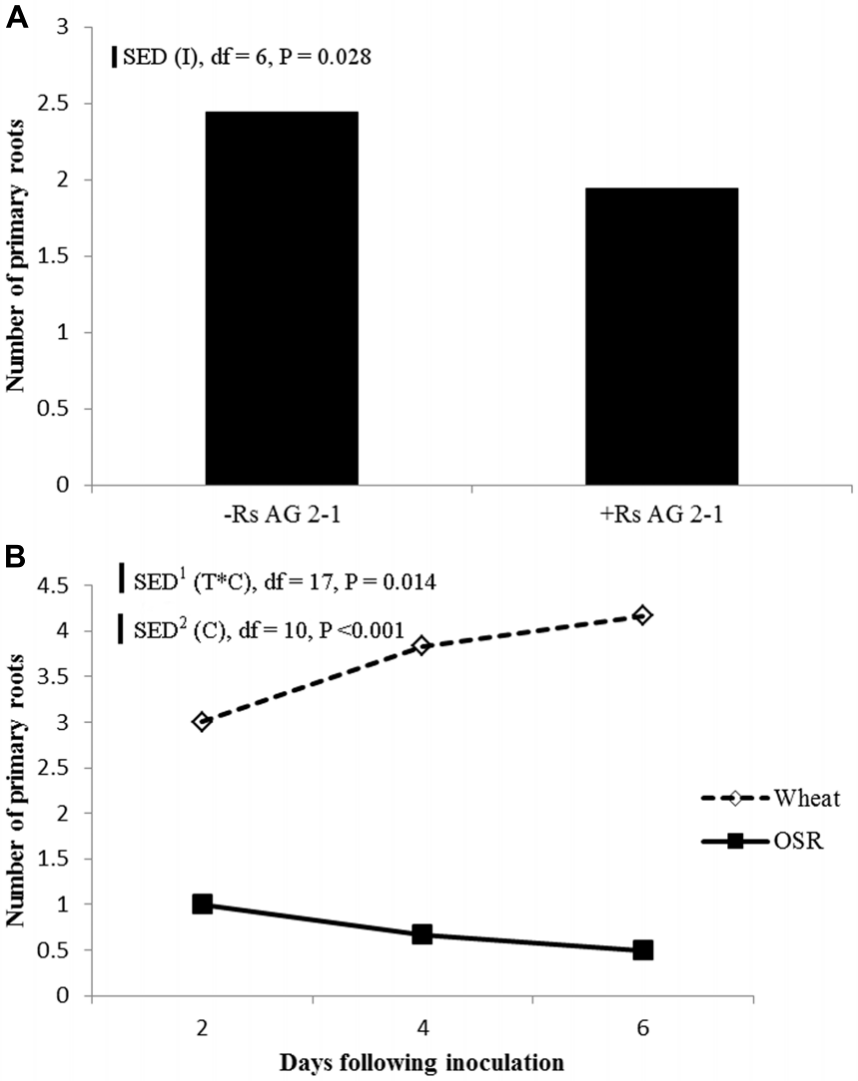
**(A)** Effect of inoculation (I) or **(B)** crop species (C), on primary root number sampled 2, 4, and 6 dfi (T) with *R. solani* AG2-1. Interactions detected using repeated measures ANOVA with degrees of freedom (df) corrected by Greenhouse–Geisser epsilon factor. Bars show SED for (1) comparing means for treatment combinations, (2) comparing means with the same level of C.

Inoculation with *R. solani* AG2-1 had a major effect on primary root number of both crops and resulted in significant reductions throughout the experiment demonstrated by the absence of significant interactions between experimental factors and time (**Figure 5A**). The number of primary roots was significantly higher in wheat compared to OSR plants which produced just one taproot (**Figure 5B**). Production of primary root numbers in wheat ceased at 4 dfi with no further significant increases being detected (**Figure 5B**). In OSR plants primary root number decreased at each sample time associated with effects of inhibition by the pathogen on root development and digestion of root tissue in time (**Figure 5B**).

Comparison of the WinRHIZO^®^ and RooTrak measurements supported all observations and displayed strong significant relationships for comparable root system traits such as volume (*P* < 0.001, *R*^2^ = 0.97) and surface area (*P* < 0.001, *R*^2^ = 0.97). The relationship for root length measured by the two methods was also significant (*P* = 0.024) but weaker than previously mentioned traits accounting for only 39% of the variance.

### Relationship of Pathogen DNA and Root System Traits

Linear regression analysis with groups for individual crops was carried out to test the fitted data for the measured traits, pathogen DNA and visual disease symptoms for position and parallelism (**Table 2**). There was a significant relationship between disease score and pathogen DNA accounting for 82% of the variance, however, the data fitted separate lines for each crop, with different slope and intercept indicating a positive relationship between pathogen DNA in soil and disease expression on plant roots for OSR only. Data fitted separate lines for each crop for root length measured by μCT on both disease (*P* < 0.001, *R*^2^ = 0.96) and pathogen DNA (*P* < 0.001, *R*^2^ = 0.77) with the same directionality showing negative relationships (**Table 2**). Similarly regressions (*P* < 0.001) of surface area and root length, measured by WinRHIZO^®^, on disease score accounted for more than 96% of the variance. Fitted separate lines with the same directionality for wheat and OSR suggested that the magnitude of effects on developing traits of the different root systems of individual crops were related to the expression of disease symptoms. All other measured traits by different systems fitted parallel lines for disease expression indicated that the final effects were similar but dependant on intrinsic differences between crops (**Table 2**).

**Table 2:** Linear regression models for disease score (y) on pathogen DNA (x) and WinRHIZO^®^, and X-ray CT based measurements of root system architecture traits (y) on disease score (x), and pathogen DNA (x) for each crop.

Dependent variable (y)	Disease score (x)			Pathogen DNA detected in soil (x)			
	*R^2^*	*P-value*	*Equation*	*R^2^*	*P-value*		*Equation*
Disease score	*	*	*	0.82	0.001	y_wheat_ = −0.014 × + 0.004	
						y_osr_ = 0.423 × + 0.018	
CT volume (mm^3^)	0.96	0.001	y_wheat_ = 0.03 ×-0.002	0.96	0.001	y_wheat_ = 0.037 × –0.00003	
			y_osr_ = 0.009 × −0.002			y_osr_ = 0.008 × –0.00003	
CT surface area (mm^2^)	0.96	0.001	y_wheat_ = 2.725 × −0.160	0.95	0.001	y_wheat_ = 2.961 × −0.002	
			y_osr_ = 0.889 × −0.160			y_osr_ = 0.779 × −0.002	
CT length (mm)	0.96	0.001	y_wheat_ = 2.644 × + 0.170	0.77	0.004	y_wheat_ = 2.436 × + 0.002	
			y_osr_ = 2.990 ×-0.578			y_osr_ = 2.727 × −0.0108	
CT convex hull (cm)	0.82	0.001	y_wheat_ = 299.3 × −12.1	0.89	0.001	y_wheat_ = 336.9 × −0.373	
			y_osr_ = 57.3 × −12.1			y_osr_ = 67.9 × −0.373	
WinRhizo volume (cm^3^)	0.93	0.001	y_wheat_ = 0.06 × −0.003	0.97	0.001	y_wheat_ = 0.07 × −0.0007	
			y_osr_ = 0.013 ×-0.003			y_osr_ = 0.013 × −0.0007	
WinRhizo surface area (cm^2^)	0.96	0.001	y_wheat_ = 4.261 × −0.956	0.97	0.001	y_wheat_ = -0.4365 × −0.004	
			y_osr_ = 1.011 × −0.202			y_osr_ = 0.993 × −0.004	
WinRhizo length (cm)	0.97	0.001	y_wheat_ = 21.269 × −4.60	0.96	0.001	y_wheat_ = 22.0 × −0.024	
			y_osr_ = 6.517 ×-1.303			y_osr_ = 5.876 × −0.024	
Primary root number	0.94	0.001	y_wheat_ = 4.242 × −0.22	0.95	0.001	y_wheat_ = 4.563 × −0.004	
			y_osr_ = 1.069 ×-0.22			y_osr_ = 0.963 × −0.004	

*Denotes values not applicable.

### Analysis of Soil Porosity

**FIGURE 6:**
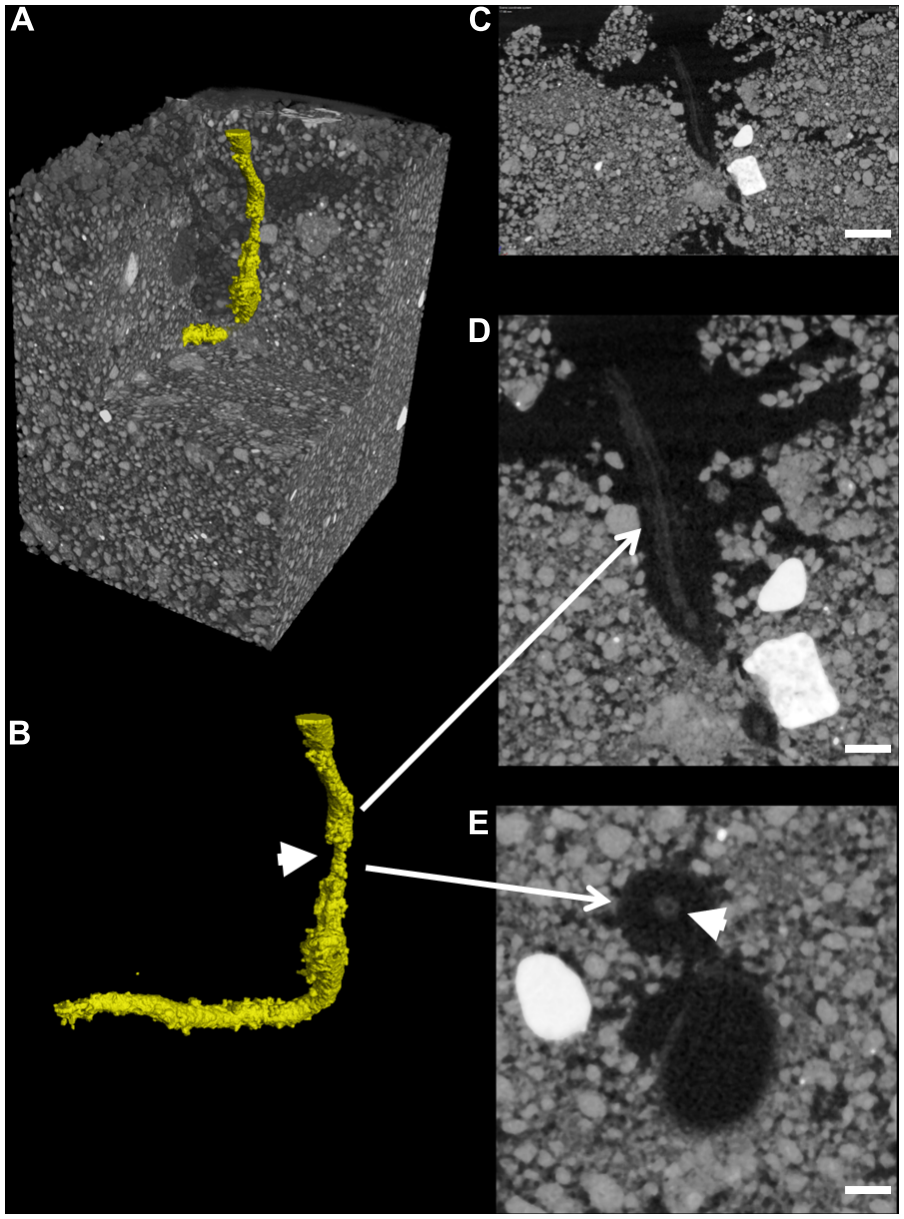
**Root cortex maceration and necrosis of developing taproot of OSR by *R. solani* AG2-1. (A)** 3D X-ray CT image of soil and root (yellow). **(B)** Image showing only root tissue (white solid arrow indicates maceration of tissue. **(C)** 2D cross-section (zx plane) image showing high porosity around OSR root (scale bar = 2 mm). **(D)** Magnified view of image shown in **(C)**, showing necrosis of root cortex (scale bar = 1 mm). **(E)** 2D cross-section (xy plane) image showing preservation of the stele (solid arrow) but complete necrosis of cortex tissue (scale bar = 0.5 mm).

Total mean soil porosity, limited to an extent by the spatial resolution of the scans, was consistent for all soil columns across all treatments (Mean, 15.4%, SEM 1.5). However, measurement of the porosity with depth within a column showed regions of variable porosity indicative of layering created during soil packing which varied between 8 and 50% (**Figure 6C**). Furthermore, there was evidence of higher porosity at the interface of the emerging seedling and the surrounding soil in some of the samples, where the highest porosity values of 50% were recorded. This was particularly evident in one of the OSR replicates treated with *R. solani* AG2-1 showing hypocotyl tissue maceration and decay in the area of high soil porosity (**Figures 6D,E**; Supplementary Video 3). However, there was only weak regression between DNA concentration and soil porosity (*R*^2^ = 0.21).

## Discussion

This work provides the first example of X-ray μCT used for the non-destructive detection of below ground symptoms and impact of *R. solani* on the developing root systems of monocotyledonous and dicotyledonous plants. *R. solani* AG2-1 causes significant pre- and post-emergence damping-off characterized by the inhibition of seed germination, root elongation, and ultimately the digestion of the root and hypocotyl of *Brassica* species (Kataria and Verma, 1992). We found moderate symptoms in OSR as early as 2 dfi and severe disease developed by 4 dfi. In contrast, only mild symptoms developed in wheat plants by 6 dfi for similar initial inoculum in the soil quantified using qPCR as pathogen DNA at 2 dfi. The difference in disease development and severity on the two crops is in agreement with previous reports on the virulence and aggressiveness of AG2-1 to OSR demonstrating that isolates belonging to this group are highly pathogenic to *Brassica* species (Gugel et al., 1987; Verma, 1996). The delay in symptom development on wheat suggests that AG2-1 is unable to cause significant symptoms on wheat confirmed by others in their investigations of pathogenicity of *R. solani* AG2-1 to cereals (Khangura et al., 1999; Oros et al., 2013). The effect of the primary host crop, OSR, on *R. solani* development was evident in the more rapid increase of pathogen DNA, reaching maximum of 300 ng g^-1^ in soil by 4 dfi in contrast to a twofold less DNA in soils from wheat grown plants (data not shown). This fast DNA accumulation in the soil from OSR, compared to wheat, is most likely related to the differences in the rate of infection and digestion of the emerging radicle and hypocotyl of the primary host species, manifested by the numerous lesions (visualized in this study) inhibited growth and ultimately the complete seedling necrosis by 6 dfi. The plateau of soil pathogen DNA at 6 adfi may be due to an exhaustion of available nutrients from the host plants and return of the pathogen to saprophytic phase of survival. The temporal dynamics of the pathogen during the development of wheat or OSR in field rotations are currently unknown. However, Brown et al. (2014) found no significant differences in pathogen DNA of *R. solani* AG2-1 accumulation in English field soils of wheat following wheat or OSR, suggesting that short wheat/OSR rotations are unlikely to be effective in reducing inoculum concentrations for either crop.

Visualization of the 3-D root system of the two crops grown in soil showed how the contrasting root systems of the monocot and dicot species reacted to the pathogen infection. Differences in the impact of the pathogen appeared to be related to the intrinsic complexity of the architectural root systems of the two crops and their ability to compensate on specific traits. Using time series μCT data importantly revealed that although the infection in the monocot, wheat, appeared asymptomatic, it contrasted the severe symptom expression in the dicot, OSR. *R. solani* AG2-1 was capable of causing significant damage on important developing root architectural traits of both crops including primary root number, root volume and root surface area that were affected less in the monocotyledonous host. Furthermore, the ability of both hosts to explore soil via their developing root system, indicated by the convex hull, was reduced. However, traits such as root length and centroid Z were not affected in the monocot. Both inoculated and control wheat plants developed 3–4 primary roots that were thicker and longer by 4 dfi compared to OSR plants. In contrast, OSR plants were mostly dependent on the development of strong taproot and subsequent lateral roots for the acquisition of resources, thus early damage to the developing taproot by *R. solani* diminished significantly the ability of the plant to establish or recover from the disease. Wheat was able to compensate by producing more than one primary root (seminal roots) and it is likely that uninfected or less severely infected roots by the pathogen were able to escape the disease and thus compensate for resource use. *R. solani* AG2-1 is most aggressive to young seedlings and host resistance to infection increases with age (Verma, 1996). Therefore faster developing OSR cultivars are more likely to escape the disease and traits related to early germination and establishment, such as seed size will be important for breeding new varieties that are more likely to tolerate *R. solani* infection (Hwang et al., 2014).

Disease score and pathogen DNA were both strongly related to changes in the measured root traits. However, the transiency of these effects in particular in the maturing wheat plant is unknown. The relationship between disease and pathogen DNA was different for the two crops and disease was only predicted successfully for OSR. This has implications in terms of assessment and prediction of disease in the field in relation to individual crop species as clear symptoms were not exhibited in wheat and not related to DNA concentrations. Furthermore, both crops suffered from *R. solani* at the seedling stage thus it is important to elucidate if the disease caused by AG2-1 is associated with significant yield loss of wheat in the field. Understanding the relationships between initial inoculum concentrations and final yield loss for the two crops can assist in the development of new strategies for prediction of risk and yield loss based on qPCR of soil prior to planting.

From the measured root traits, only root length showed poor correlation between the two imaging approaches which can be attributed to the way the trait was measured. RooTrak root length measurements were limited to a maximum soil depth of 25.80 mm compared to the entire 30 mm column length due to the field of view possible in a single μCT scan. However, as RooTrak can quantify novel root traits such as convex hull, there is potential to measure crop species specific descriptors to define root structure, e.g., differences between the single tap root of OSR versus primary and seminal root system of wheat. A crucial advantage of the μCT imaging is that not only can the developing root systems be quantified non-destructively and temporally but as we have shown changes in the soil microstructure can also be considered. Although, our initial soil conditions were designed as in most repacked column studies to be uniform, verification of the microstructure by imaging showed localized variations in porosity when measured at high resolutions especially at the root surface. This zone, i.e., the rhizosphere, is a crucial interface, where knowledge about the structural arrangement in particular is lacking. Variations in structure as we have revealed here will influence soil moisture availability considering the relationship between matric suction and pore size. Soil bulk density and moisture content are known to significantly influence hyphal growth and disease severity caused by *R. solani* (Glenn et al., 1987; Gill et al., 2001, 2004) but the impact at the pore scale is less well understood. Furthermore, it is generally accepted that the key limiting factor in hyphal proliferation is the availability of air filled pores within the soil (Glenn et al., 1987; Otten et al., 1999; Harris et al., 2003). We found OSR seedlings displaying the highest porosity around the seedling also had the lowest disease severity and longest root and shoot growth (**Figure 6**). This finding is in agreement with Gill et al. (2000) who found that although saprotrophic growth was higher in more porous soils, the disease severity was lower highlighting the potential of X-ray μCT in the study of the physical effects of soil structure on soil-borne pathogenic fungal diseases. This has potential implications for soil management practices, such as conventional and zero tillage as these may have very different soil structures (Mangalassery et al., 2014). For example, plowing could potentially reduce soil-borne disease severity, by increasing the porous structure of soil, physical disruption to fungal hyphal networks and increasing background microbial activity. Indeed, the most effective cultural control method for soil-borne *Rhizoctonia* root patch in wheat is via tillage practice of soil disturbance by cultivation which destroys established fungal hyphal networks and can increase microbial activity (Paulitz et al., 2002). The effect of tillage on soil-borne pathogens in OSR has received less attention, however, it is likely that reduced or zero tillage maximizes disease and inoculum potential by allowing infected crop residues to remain on the soil surface and preserving hyphal networks in close proximity to the host (Kharbanda and Tewari, 1996). Although soil structure can routinely be imaged at high resolutions (i.e., <100 μm), it is still not possible to visualize fungi *per se* using X-ray μCT due to their very low X-ray attenuation (Gleason et al., 2012). However, indirect modeling approaches have been useful to aid understanding of the behavior and functioning of fungi in both real (Pajor et al., 2010; Falconer et al., 2011) and artificial soil microstructures (Otten et al., 2012). These combined approaches may be of value in the future to facilitate further understanding of plant pathogenic fungi in the soil environment.

This study has successfully quantified the impact of *R. solani* on crop root system traits and development through the combined use of X-ray μCT and qPCR. X-ray μCT offers more promise than destructive methods as the development of disease symptoms on the root can be monitored non-destructively in soil. We have shown that disease symptoms developed rapidly in OSR within 2 dfi, whereas wheat displayed a higher tolerance with only mild symptoms present after 6 dfi. Differences in the impact of the pathogen on the two hosts were related to complexity and developmental rates of the different root architectural types of the monocot, wheat, and the dicot, OSR.

## Conflict of Interest Statement

The authors declare that the research was conducted in the absence of any commercial or financial relationships that could be construed as a potential conflict of interest.
